# Single-cell sequencing combined with machine learning to identify glioma biomarkers and therapeutic targets

**DOI:** 10.3389/fonc.2025.1629102

**Published:** 2025-07-24

**Authors:** Yu Yan, Zhengmin Chu, Qi Zhong, Genghuan Wang

**Affiliations:** Department of Neurosurgery, The Second Affiliated Hospital of Jiaxing University, Zhejiang, China

**Keywords:** Glioma, ScRNA-seq, prognosis, biomarker, machine learning

## Abstract

**Background:**

The purpose of this study is to utilize single-cell sequencing data to explore glioma heterogeneity and identify key biomarkers associated with glioblastoma multiforme (GBM) relapse using machine learning.

**Methods:**

Single-cell sequencing and transcriptome data for gliomas were obtained from the GEO (GSE159416, GSE159605, and GSE186057) and TCGA databases. A prognostic model based on differentiation-related genes (DRGs) was constructed using weighted correlation network analysis, univariate Cox regression, and LASSO analysis. Key genes were identified using LASSO and SVM-RFE, with intersecting genes selected as the final set of key genes. Further analyses examined immune infiltration patterns and functional pathways. Importantly, we analyzed the relationship between prognostic-related genes and ubiquitination, and further characterized the characteristics of ubiquitination-related prognostic genes. In addition, we performed CCK-8 assays, colony formation, Transwell invasion assays, apoptosis assays to determine the role of ETV4 in glioma.

**Results:**

Examination of single-cell RNA-seq data from the GEO database revealed three distinct cell differentiation stages in glioma tissues. Marker genes for each of these cell states were combined to form DRGs. A 16-gene DRG signature was developed for predicting the survival of glioma patients. Machine learning identified four important genes with high AUCs in both training and test sets. Notably, 13 out of 16 genes in the DRG signature are ubiquitin-related, highlighting the involvement of ubiquitination in GBM. Moreover, we reported that inhibition of ETV4 attenuates cell proliferation and invasion in glioma cells.

**Conclusion:**

Our prognostic model, based on the differentiation-related gene signatures, may be valuable for predicting prognosis and immunotherapy response in glioma patients. Characterizing these ubiquitination-associated features may elucidate the molecular mechanisms driving GBM progression and offer novel insights for its diagnosis and treatment. Additionally, machine learning identified four biomarkers with potential for aiding in the diagnosis and treatment of GBM.

## 1 Introduction

Glioblastoma Multiforme (GBM) is the most common and aggressive primary brain tumor, characterized by invasive growth and resistance to conventional treatments (1, 2). The five-year survival rate for patients with GBM is only 7%, highlighting the poor prognosis associated with this disease (3). The World Health Organization (WHO) classifies gliomas into four grades, with high-grade gliomas (WHO grade 3 to 4) often exhibiting poor prognoses (4). For patients with GBM (WHO grade 4), median survival ranges from 14.6 to 17 months (5).

The complexity of GBM treatment is complicated by tumor heterogeneity, arising from the diverse types of GBM cells and a complex tumor microenvironment (6–8). This heterogeneity exists not only between individuals but also within individual tumors (9, 10). Single-cell sequencing (SCS) has been reported to explore the mechanisms of disease development (11, 12). SCS allows researchers to examine interactions between various types of GBM cells and neoplastic cells at a more detailed level (13). Unlike single-cell sequencing, next-generation sequencing (NGS), which is commonly used, analyzes the entire cell population and is unable to capture cellular heterogeneity (14). SCS amplifies and sequences the genome or transcriptome at the single-cell level, providing information on single nucleotide variations (SNVs), gene copy number variations (CNVs), single-cell genome structure variations, gene expression, gene fusions, alternative splicing in the single-cell transcriptome, and DNA methylation in the single-cell epigenome (15). SCS enables the study of genetic characteristics in diseases and biological processes at the single-cell level, including early embryonic development, tumorigenesis mechanism, tumor heterogeneity and evolution, as well as circulating tumor cells (CTCs) and clonal evolution (16). Meanwhile machine learning brings new opportunities for identifying biomarkers (17). By efficiently filtering out irrelevant features, machine learning is highly suitable as a tool for pre-screening features (18, 19).

Protein post-translational modifications (PTMs) are covalent and enzymatic alterations that occur during or after biosynthesis, modulating protein properties and functions. Among them, non-histone PTMs, including acetylation, lactylation, methylation, ubiquitination, phosphorylation, and SUMOylation, have been reported to be closely associated with cancer progression (20). Abnormalities in PTMs have been observed to influence cancer cell proliferation, migration, and invasion. Ubiquitination, a crucial PTM, governs protein stability and a wide range of cellular processes by attachment of ubiquitin molecules (21). Emerging evidence suggests that ubiquitination plays a pivotal role in mediating resistance to cancer immunotherapy (22). For instance, the ubiquitin-conjugating enzyme E2S has been reported to reduce the sensitivity of GBM cells to temozolomide by upregulating PGAM1 through interaction with OTUB2 (23). In addition, centromere protein U facilitates temozolomide resistance by mediating the ubiquitination and degradation of RPS3 in GBM (24). RNF8-mediated ubiquitination of KRT80 has been shown to drive glucose metabolic reprogramming and GBM progression (25). Moreover, the tryptophan-metabolizing enzyme IL4I1 inhibits ferroptosis in GBM by decreasing the ubiquitination of Nrf2 via I3P (26). Therefore, ubiquitination is critically involved in GBM development and progression.

This study aims to analyze glioma single-cell RNA sequencing (scRNA-seq) data to explore heterogeneity of GBMs and identify differentiation-related genes (DRGs) for prognostic prediction using bulk RNA-seq data. Additionally, support vector machines recursive feature elimination (SVM-RFE) and least absolute shrinkage and selection operator (LASSO) machine learning algorithms were employed to select key biomarkers associated with GBMs and enhance predictive accuracy for GBM prognosis. Importantly, this study also investigated transcription factors, protein-protein interaction networks, enriched pathways and biological functions, differential expression patterns, and survival associations of ubiquitin-related prognostic genes.

## 2 Methods

### 2.1 Data collection

Single-cell sequencing data were obtained from the GEO database (Gene Expression Omnbius) under accession GSE159416 (27) (https://www.ncbi.nlm.nih.gov/geo/), which includes single-cell RNA-seq data from 18 glioma patients. Additionally, clinical data and gene expression data were sourced from the TCGA (The Cancer Genome Atlas, https://www.cancer.gov/about-nci/organization/ccg/research/structural-genomics/tcga). Array-based expression profiles were collected from GEO datasets GSE159605 and GSE186057. A total of 1041 samples were included in this study.

### 2.2 Quality control for SCS data processing

ScRNA-seq data were preprocessed using “Seurat” and “Monocle” R packages. The PercentageFeatureSet function was used to calculate the number of mitochondrial genes, and cells with less than 500 or more than 5000 were removed based on quality control results. The LogNormalize method was used to normalize scRNA-seq data, and the ‘vst’ selection method was used to identify the top 1000 highly variable genes. Principal component analysis (PCA) was used to reduce dimensions for glioma cells. Using the t-distributed stochastic neighbor embedding (tSNE) approach, the top 10 principal components (PCs) with significant values were chosen for clustering. The ‘limma’ package was used to find marker genes in each cluster, selecting those with an adjusted p value of less than 0.05 and a |Log2 fold change (FC)| > 2 value. ScRNA-seq data were automatically annotated using the “SingleR” tool. Data from the “celldex” package’s major human cell atlas were utilized as reference data.

### 2.3 Pathway and pseudotime analysis

For astrocyte and tissue stem cells, pseudotime and trajectory analyses were carried out using the “Monocle” package, with the distribution of cells along each branch representing a single differentiation condition. |log2 (FC)|> 2 and adjusted p- values < 0.05 were used to identify DEGs in cells that were in separate development states. GO (Gene ontology) analysis, which categorizes gene functions, was applied to differentially expressed transcripts to provide a structural functional description. In the transcriptome project, GO functional analysis gives a categorical annotation of GO function for differentially expressed transcripts. ClusterProfiler, org.Hs.eg.db, enrichplot, and ggplot2 packages were used to analyze the KEGG (Kyoto Encyclopedia of Genes and Genomes, https://www.kegg.jp/kegg/kegg1.html) pathways and GO for DRGs.

### 2.4 Consensus clustering and prognosis analysis

Consensus clustering, an unsupervised clustering method, was used to distinguish samples into several subtypes according to different omics datasets. Data were classified using the “ConsensusClusterPlus” R package. Kaplan-Meier analysis was conducted to compare overall survival across different clusters, and a prognostic modle was developed using Cox regression analysis.

### 2.5 Ubiquitination−related prognosis genes

A list of Ubiquitination-related genes (URGs) was obtained by querying the human gene database GeneCards (https://www.genecards.org/) using the keyword “Ubiquitination” (28). The “venn” R package was then utilized to identify the intersection between URGs list and glioma-associated prognostic genes (GAPGs). Transcription factor analysis was conducted using the transcriptional regulatory relationships unraveled by sentence-based text mining (TRRUST, version 2, https://www.grnpedia.org/trrust/Network_search_form.php), which employs sentence-based text mining to uncover transcriptional regulatory relationships (29). Protein-protein interaction (PPI) networks were constructed using the STRING database (Version 12.0, https://cn.string-db.org/) (30). Functional annotation and enrichment analyses of gene lists were performed using Metascape (https://metascape.org/gp/index.html#/main/step1) (31). Additionally, differential gene expression and survival analyses were carried out using the GEPIA database (http://gepia.cancer-pku.cn/), which integrates tumor and normal tissue data from TCGA and GTEx (32).

### 2.5 Machine learning

Distinctive genes were screened using SVM-RFE (33) and LASSO (34). Key genes were individually filtered by LASSO and SVM-RFE, and the intersecting genes were then chosen as the final key genes. LASSO reduced model complexity by selectively incorporating variables, optimizing performance while controlling complexity to avoid overfitting. SVM-RFE provides high-accuracy feature screening and uses incremental regularization to prevent overfitting. Both algorithms were employed to screen for prognostic genes, and the intersecting genes from both methods were chosen as the final prognostic markers.

### 2.6 WGCNA

Weighted correlation network analysis (WGCNA) is a systems biology approach that identifies highly correlated gene sets and potential biomarkers by analyzing the association between gene modules and phenotype genes or therapeutic targets. WGCNA classifies co-expressed genes into modules, facilitating investigation of module-phenotype relationships. In this study, WGCNA was used to identify genes linked to the LLPS phenotype. The pickSoftThreshold function from the “WGCNA” R package was used to determine the optimal soft field value.

### 2.7 Immune inflation and immune checkpoint analysis

The “cibersort” package (35) was used to quantify related infiltration and activity levels for 22 immune cell types based on published gene signature lists across all tumors and normal samples. Immune cells types in this study included activated dendritic cells (DCs), neutrophils, mast cells, eosinophils, macrophages, and components of adaptive immunity such as B cells, T cells, central memory T cells. A heatmap was created to represent the findings of the investigation on the immunological checkpoint differences between high- and low- risk groups.

### 2.7 Cell culture and transfection

The human glioma cell lines LN229 and U251 were obtained from the Shanghai Cell Bank (Shanghai, China). Cells were cultured in Dulbecco’s Modified Eagle Medium (DMEM) supplemented with 10% fetal bovine serum (FBS) and 1% penicillin-streptomycin. Cells were maintained in a humidified incubator at 37°C with 5% CO_2_.

Short hairpin RNA (shRNA) targeting ETV4 gene and shRNA control (shNC) were purchased from GenePharma (Shanghai, China) company and transfected into LN229 and U251 cells using Lipofectamine 3000 (Invitrogen) according to the manufacturer’s protocol. shETV4-1: GCT GGA TGA CCC AAC AAA T; shETV4-2: CCC TGT GTA CAT ATA AAT GAA. Knockdown efficiency was validated by Western blotting.

### 2.8 Western blotting analysis

Cells were lysed in RIPA buffer (Beyotime, China) and protein concentrations were determined using the BCA Protein Assay Kit. Protein were separated by SDS-PAGE and transferred onto PVDF membranes. Membranes were blocked with 5% non-fat milk for 1 hour at room temperature, then incubated with anti-ETV4 antibody (CST #65763) overnight at 4°C. Protein bands were visualized using an ECL detection kit as described previously.

### 2.9 CCK-8 assays and colony formation

Cell viability was assessed using the Cell Counting Kit-8 (CCK-8) according to the manufacturer’s instructions. Briefly, transfected cells were seeded into 96-well plates. After the specified durations, 10 µL of CCK-8 reagent was added to each well. After cells were incubated at 37°C for 2 hours. Absorbance was measured at 450 nm by a microplate reader. Transfected cells were seeded into 6-well plates and cultured in complete medium for 14 days, allowing colonies to form. Then, cells were fixed with 4% paraformaldehyde for 15 minutes and stained with 0.1% crystal violet for 30 minutes at room temperature. After washing with PBS, colonies were imaged and counted.

### 2.10 Cell apoptosis assays

Cell apoptosis was evaluated using an Annexin V-FITC/PI Apoptosis Detection Kit according to the manufacturer’s instructions. Briefly, cells were harvested, washed, and resuspended in binding buffer. Then, 100 μL of the cell suspension was incubated with 5 μL Annexin V-FITC and 5 μL propidium iodide (PI) for 15 minutes at room temperature in the dark. After incubation, samples were analyzed using a flow cytometer. Cells were classified as viable, early apoptotic, late apoptotic, and necrotic.

### 2.11 Cell invasion assays

Cell invasion was assessed using Transwell chambers (Corning, USA) coated with Matrigel. Briefly, Cells were suspended in 200 μL serum-free medium and seeded into the upper chamber. The lower chamber was filled with medium containing 10% FBS. After incubation at 37°C for 24 hours, non-invading cells on the upper surface were removed with a cotton swab, and the invading cells on the lower surface were fixed with 4% paraformaldehyde and stained with Calcein AM. Invaded cells were imaged and counted under a microscope.

### 2.12 Statistical analysis

All statistical analyses were calculated using R software version 4.1.3 and the corresponding packages. Data were analyzed using student t-tests and one-way ANOVA for *in vitro* experiments. p < 0.05 was considered statistically significant.

## 3 Results

### 3.1 Single-cell sequencing analysis

**Figure 1:**
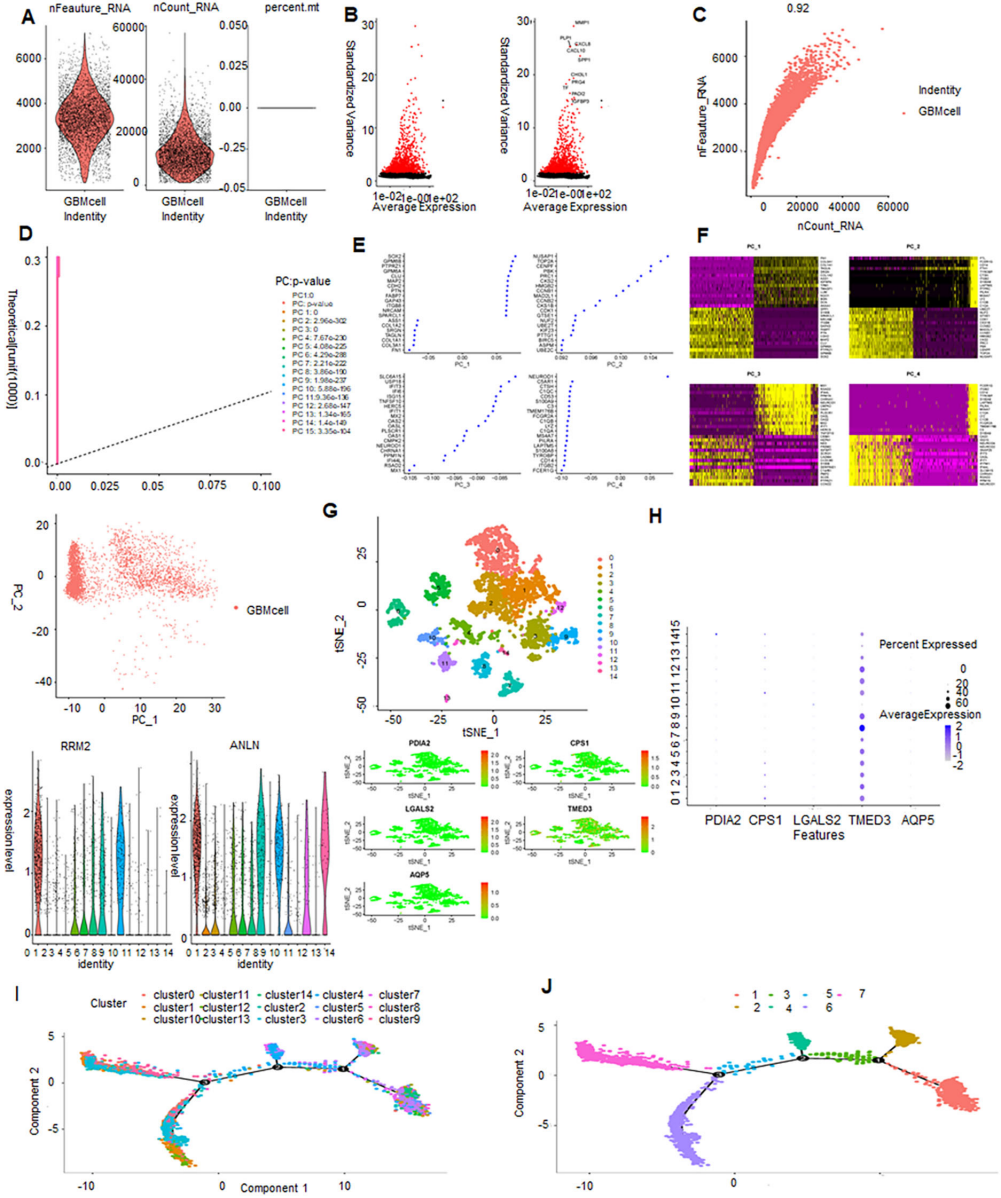
Single-cell sequencing analysis: quality control, dimensionality reduction, clustering, gene expression, and trajectory analysis. **(A, B)** Violin plot displaying the results of quality control and filtration for the scRNA-seq dada. **(C)** Scatter plot showing the correlation between sequencing depth and the number of detected genes. **(D)** PCA plot represents single-cell distribution in a reduced-dimensional space through principal component analysis (PCA). **(E)** Top 20 genes plotted for each principal component (PC). **(F)** Heatmap of the top 20 genes for PC, where each row represents a gene, and each column represents a sample or condition. Darker colors indicate higher expression levels. **(G)** Scatter plot of cell clusters by t-SNE: visualizing cellular heterogeneity and clustering patterns. **(H)** Expression levels of PDIA2, PS1, GALS2, MED3, QP5 in 14 clusters. **(I)** Trajectory analysis of 14 clusters. **(J)** Trajectory analysis of epithelial cells and macrophages.

The ranges of single-cell RNA counts, as well as RNA counts per cell, demonstrated a high level of sample quality control (**Figure 1A**). The 2000 most variable genes and the top 10 genes identified across all samples are displayed (**Figure 1B**). The number of identified genes and sequencing death showed a strong positive association (R = 0.92, **Figure 1C**). Next, we performed PCA on the normalized cell data (**Figure 1D**). Scatterplots were generated to depict the relationship between PC scores and selected genes, where larger absolute score values indicate stronger correlations. For each PC, the top 20 genes were chosen based on their coefficients. In PC_1, genes SOX2 and GPM6B exhibited significant correlations with score values (**Figure 1E**).

A gene expression heatmap was generated to visualize the expression levels of the top 20 selected genes for each PC, where darker colors indicate higher expression levels (**Figure 1F**). To investigate the relationship and clustering patterns among all cells, the t-distributed stochastic neighbor embedding (t-SNE) algorithm was employed. This algorithm effectively reduces high-dimensional data to a two-dimensional space. This analysis identified 14 distinct clusters, with cells closer together in the t-SNE plot indicating greater similarity (**Figure 1G**). To validate the optimal clustering results and identify potential feature genes for each subgroup, the top 20 genes for each cellular subgroup were generated, including ANLN, RRM2, PDIA2, CPS1, LGALS2, TMED3, and AQP5.

**Figure 2:**
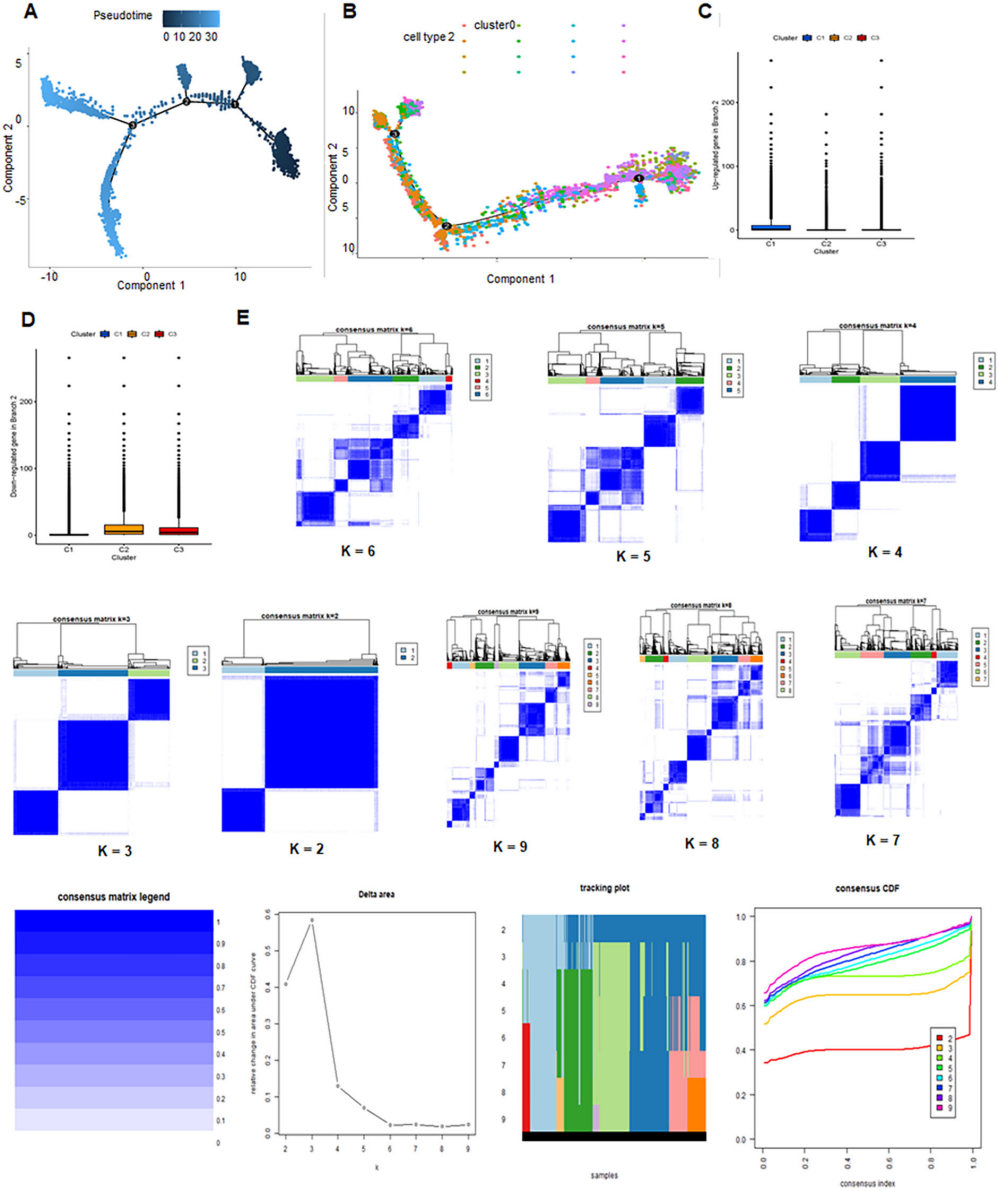
Developmental trajectories and consensus clustering in glioblastoma multiforme (GBM). **(A, B)** Trajectory analysis of 14 clusters unveiling developmental paths and cellular dynamics. **(C, D)** Expression of upregulated and downregulated DRGs in C1, C2 and C3 clusters. **(E)** Consensus clustering analysis for GBM.

TMED3 exhibited high expression across all subclusters, indicating a ubiquitous expression pattern within the analyzed dataset (**Figure 1H**). Pseudo-temporal analysis was used to analyze cellular motion trajectories across the 14 subclusters, inferring the temporal progression and cell movement dynamics within each subcluster (**Figure 1I**). Cell type annotation was performed on cells distributed into 14 clusters based on marker gene expression. Additionally, pseudo-temporal analysis on the annotated cells inferred their temporal order or developmental trajectory (**Figure 1J**). A total of 17,846 astrocytes and stem cells underwent pseudotime and differentiation trajectory analysis (**Figures 2A, B**), revealing 3 branches of cells with diverse differentiation patterns. Cells in state 1 were inferred as the initial cell type, subsequently differentiating into various states (**Figures 2C, D**).

### 3.2 Consensus clustering

Consensus clustering was performed on cell populations to classify cells. The results suggested that the optimal clustering occurred at K = 2. The delta area plot indicated the best K value by identifying the point with the least increase in stability. **Figure 2** showed the sample clusters at each K value with a heatmap, which qualitatively assesses unstable clusters and samples (**Figure 2E**).

### 3.3 KEGG and GO analysis

**Figure 3:**
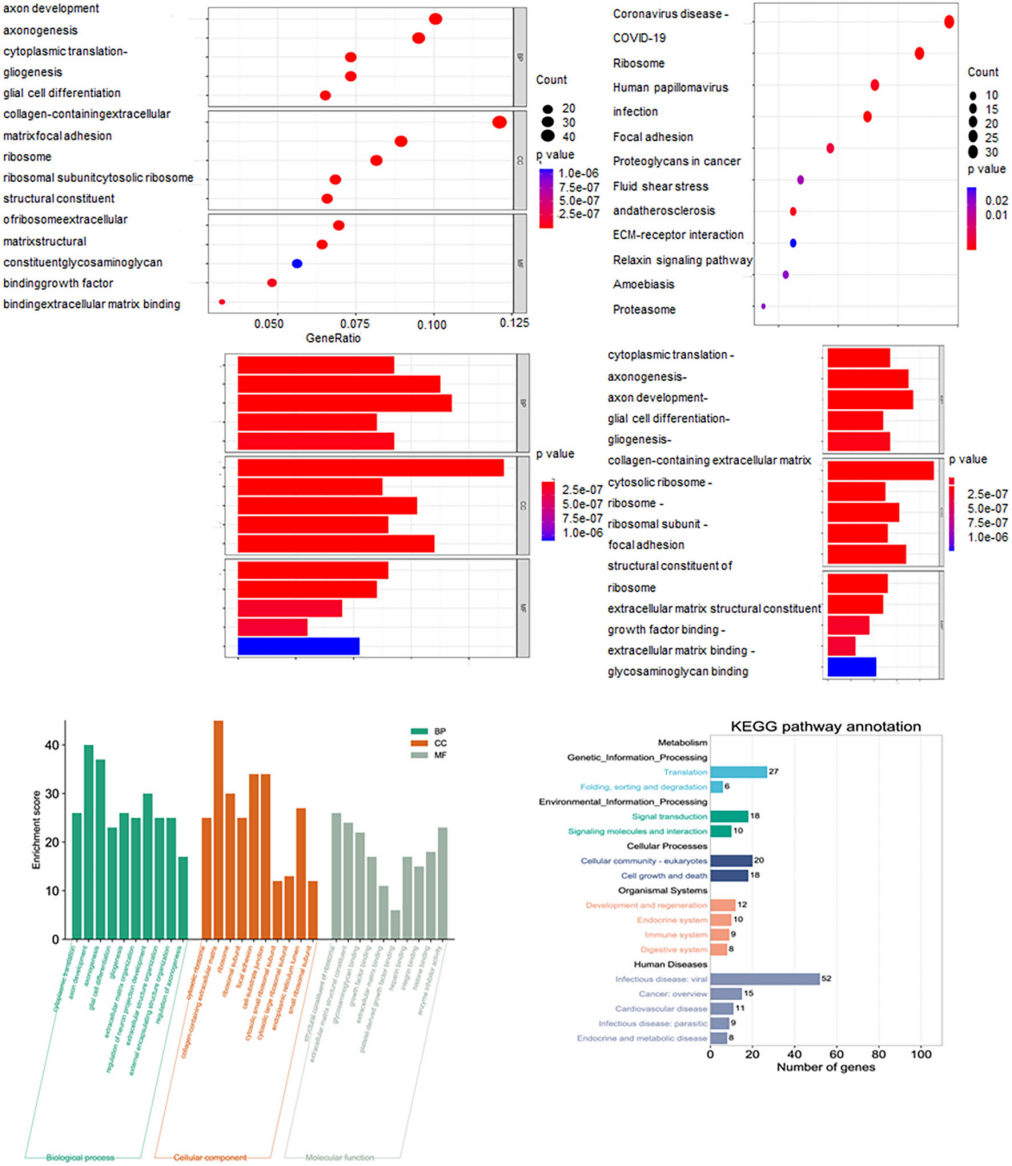
KEGG and GO analysis uncovering functional enrichment and pathway insights.

KEGG pathway analysis indicated that GBM development is associated with pathways including Coronavirus disease (COVID-19), ribosome, human papillomavirus infection, focal adhesion, proteoglycans in cancer, shear stress and atherosclerosism, ECM-receptor interaction, relaxin signaling pathway, amoebiasis and proteasome. GO analysis suggested that the molecular functions of the genes may relate to cytoplasmic translation, axonogenesis, axon development, glial cell differentiation and gliogenesis (**Figure 3**).

### 3.4 Immune infiltration and immune checkpoint analysis

**Figure 4:**
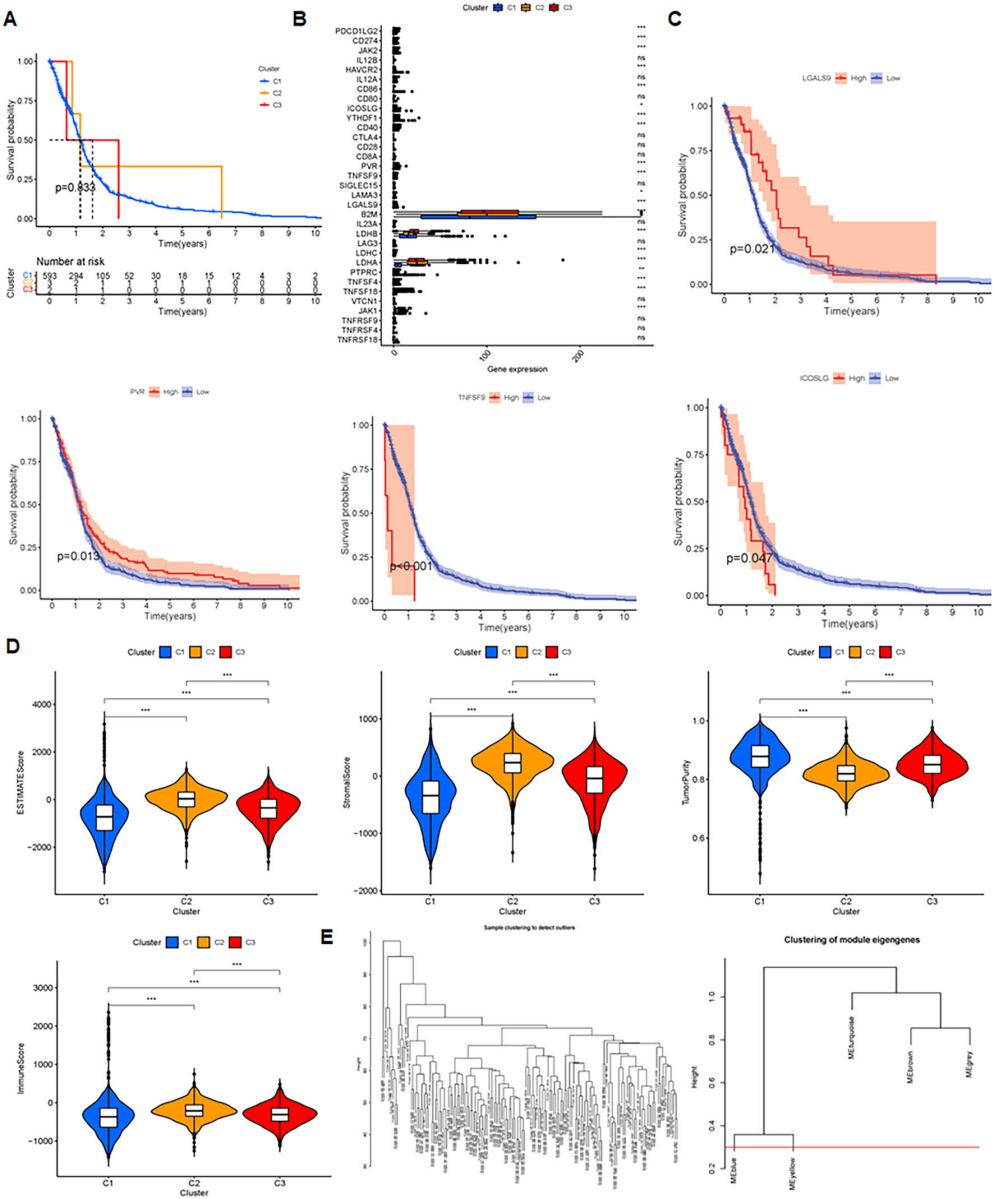
Insights and analysis for prognosis, immune checkpoints, and clustering. **(A)** Prognosis analysis of 3 clusters evaluating survival outcomes and predictive significance. **(B)** Immune checkpoint analysis in C1, C2 and C3 clusters. ‘ns’ means no significance, *P < 0.05, **P < 0.01, and ***P < 0.001. **(C)** Prognosis related to four checkpoints, assessing the impact of checkpoint expression on patient survival. **(D)** Comparisons of immune score, stromal score, ESTIMATE score, and tumor purity, examining tumor microenvironment and predictive metrics. **(E)** WGCNA clustering uncovering co-expression patterns and functional modules.

Prognostic analysis revealed no significant differences among clusters 1, 2, and 3 (p = 0.833) (**Figure 4A**). However, immune checkpoints PDC1LG2, CD274, JAK2, HAVCR2, CD86, ICOSLG, YTHDF1, CD40, PVR, and TNFSF9 showed significant differential expression across clusters (**Figure 4B**). Glioma prognosis was correlated to immune checkpoints LGALS9, PVR, TNFSF9 and ICOSLG (**Figure 4C**). Cluster C2 samples had the highest immune, stromal, and ESTIMATE scores, but the lowest tumor purity (p < 0.001), showing the highest quantity of immune and stromal cells. In contrast, C1 had the highest tumor purity and the least amount of immune and stromal cells (**Figure 4D**). Moreover, WGCNA clustering showed co-expression patterns and functional modules (**Figure 4E**).

### 3.5 Weighted gene co-expression network analysis

**Figure 5:**
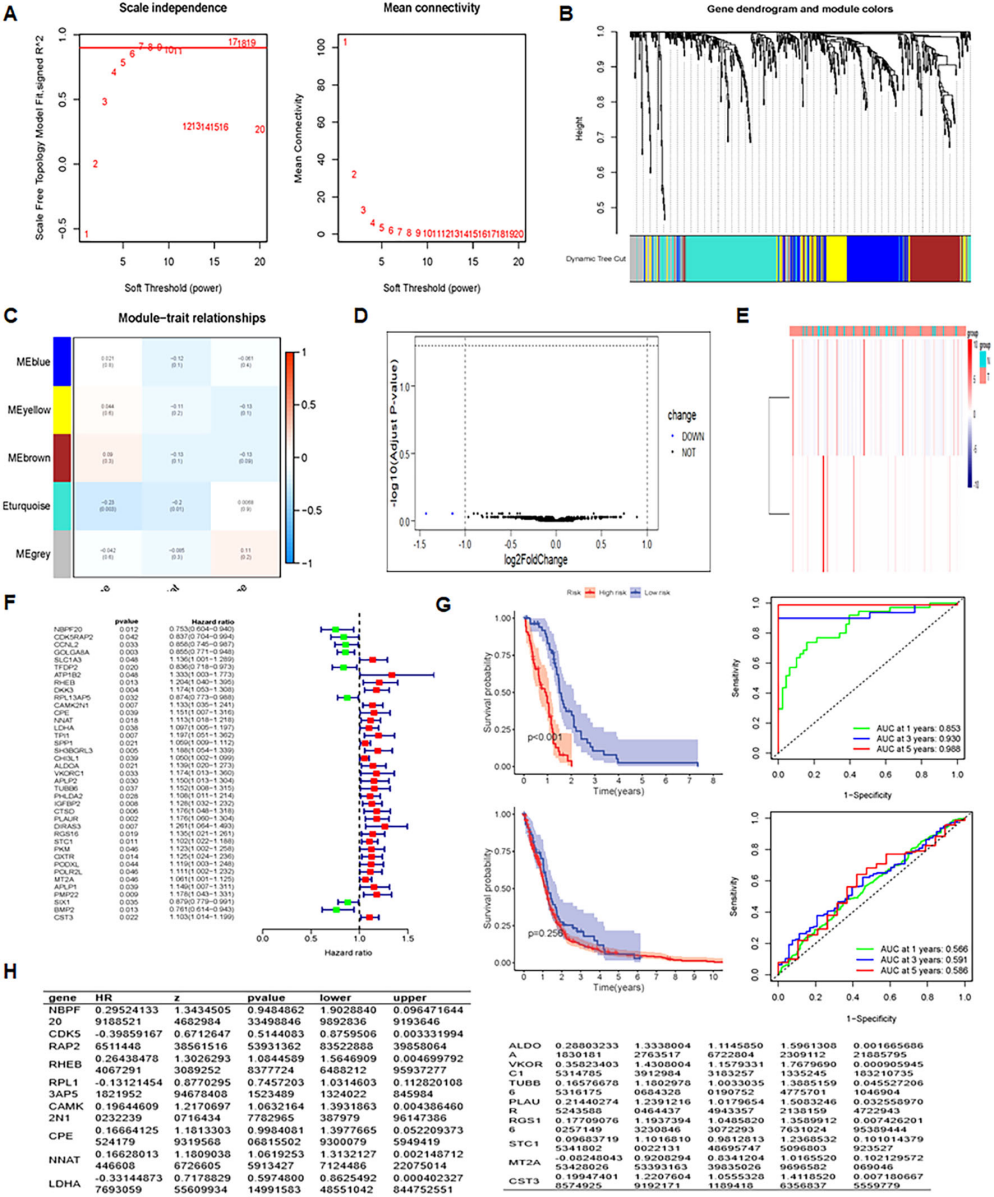
Integrative analysis and insights unveiling prognostic factors, differential expression, and regression results. **(A)** Analysis of scale-free fit index and mean connectivity assessing the fit of network modules at different soft-thresholding powers. **(B)** Dendrogram of differentially expressed genes, clustering based on dissimilarity measure (1-TOM). **(C)** Heatmap of module eigengene-clinical status correlation: showing relationships between module eigengenes and clinical status (Normal and Tumor). **(D)** Volcano plot of differentially expressed genes, visualizing statistical significance and fold change. **(E)** Heatmap of differentially expressed genes, illustrating gene expression patterns and clustering. **(F)** UniCox regression analysis results, evaluating gene expression associations with survival. **(G)** Prognosis and AUC analysis, assessing prognostic performance in training and test groups.

WGCNA was employed to identify co-expressed gene modules and explore association between the gene network, phenotype, and core genes (36). Analysis was feasible with the soft domain value set to 6 (**Figure 5A**). WGCNA organized genes into modules, ultimately categorizing glioma gene expression into five distinct modules (**Figure 5B**). Mean connectivity remained constant when the soft threshold increased. All genes were grouped into 15 modules (**Figure 5C**), and the ME yellow module showed the strongest correlation with the survival time (futime) (**Figure 5D**, P < 0.05). Differential expression analysis on the key genes from the key modules identified two downregulated genes, while the remaining genes showed no differential expression (**Figure 5E**). The heatmap of differential expression is displayed in **Figure 5E**.

### 3.6 Prognosis model of GBM

**Figure 6:**
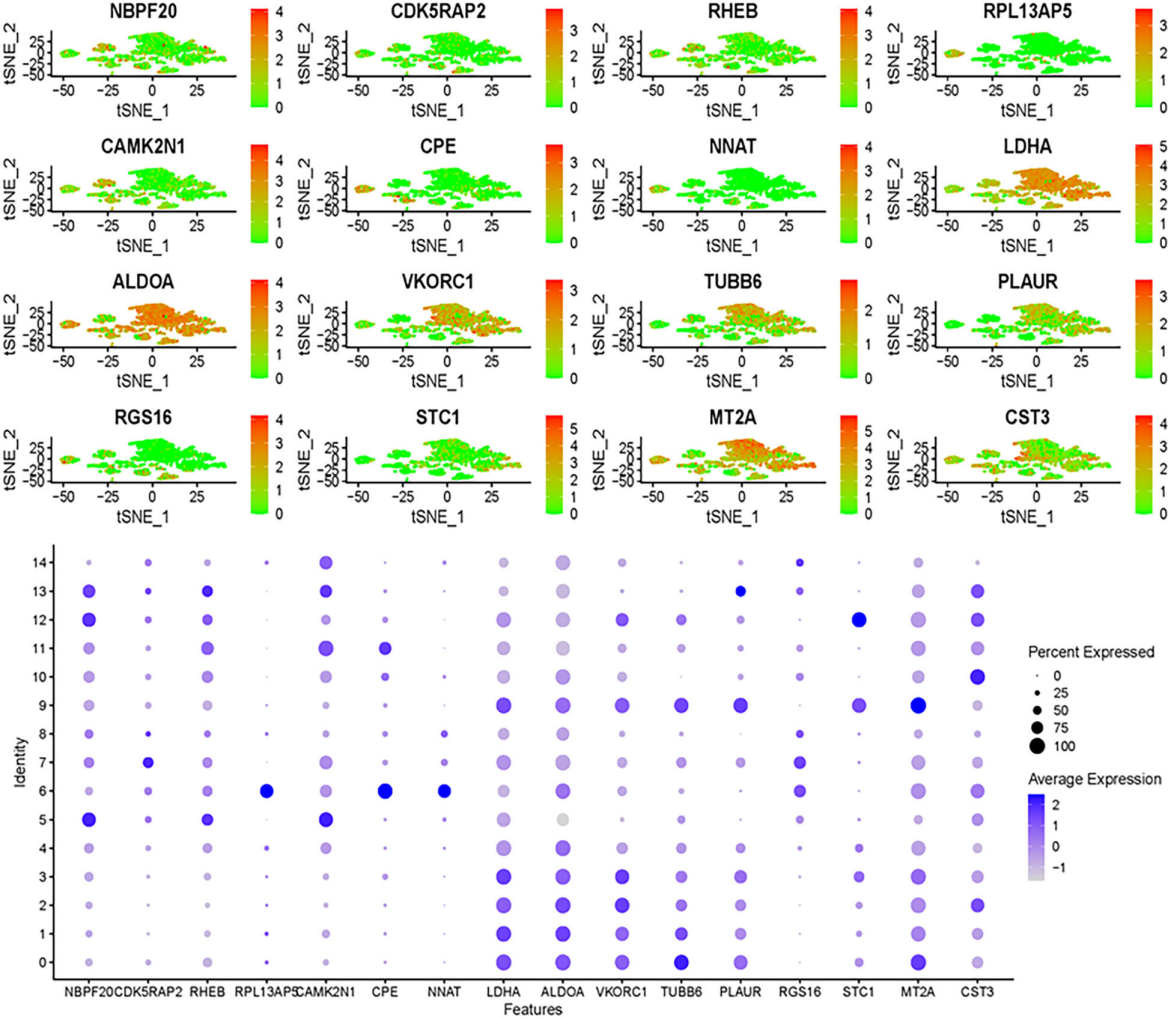
Gene expression analysis using single-cell data uncovers expression patterns of genes identified through multi-Cox regression.

A prognostic model for glioma was constructed using multiple Cox regression analysis of 532 genes screened from WGCNA. The LASSO algorithm was used to prevent overfitting. Univariate Cox regression analysis narrowed down 39 genes, followed by multiple distant linear regression (**Figure 5F**), resulting in 16 glioma-associated prognostic genes for the model (**Table 1**). Data were randomly split into training and test datasets and further divided into high-risk and low-risk groups based on the median risk score. In the training dataset, low-risk group patients had a significantly better prognosis than those in the high-risk group. However, in the test group, no significant difference in survival prognosis was observed between the two groups. The AUC values for the training dataset were 0.853 at one year, 0.930 at three years, and 0.968 at five years, which implies an improvement in predictive ability over time. For the test dataset, AUC values were 0.566 at one year, 0.591 at three years, and 0.586 at five years (**Figure 5G**). The 12 genes in the model were put into the single-cell data to assess their enrichment. Results demonstrated differential expression of the 12 genes across enriched clusters. Notably, NBP20 was primarily expressed in clusters 5, 12 and 13. RPL13 and CDK5 were significantly expressed in clusters 7 and 6, respectively. Additionally, the gene expression of LDHA, ALDOA, VKORC1, TUBB6, and PLAUR was enhanced across clusters (**Figure 6**).

**Table 1:** 16 glioma-associated prognostic genes.

Gene	HR	Z	P value	Lower	Upper
NBPF20	0.295241339188521	1.34345054682984	0.948486233498846	1.90288409892836	0.0964716449193646
CDK5RAP2	-0.398591676511448	0.671264738561516	0.514408353931362	0.875950683522888	0.00333199439858064
RHEB	0.264384784067291	1.30262933089252	1.08445898377724	1.56469096488212	0.00469979295937277
RPL13AP5	-0.131214541821952	0.877029594678408	0.74572031523489	1.03146031324022	0.112820108845984
CAMK2N1	0.196446090232239	1.21706970716434	1.06321647782965	1.3931863387979	0.00438646096147386
CPE	0.16664125524179	1.18133039319568	0.998408106815502	1.39776659300079	0.0522093735949419
NNAT	0.16628013446608	1.18090386726605	1.06192535913427	1.31321277124486	0.00214871222075014
LDHA	-0.331448737693059	0.717882955609934	0.597480014991583	0.862549248551042	0.000402327844752551
ALDOA	0.288032331830181	1.33380042763517	1.11458506722804	1.59613082309112	0.00166568621885795
VKORC1	0.358234035314785	1.43080043912984	1.15793313183257	1.76796901335245	0.000905945183210735
TUBB6	0.165766785316175	1.18029780684328	1.00330350190752	1.38851594775701	0.0455272061046904
PLAUR	0.214402745243588	1.23912160464437	1.01796544943357	1.50832462138159	0.0325589704722943
RGS16	0.177090760257149	1.19373943230846	1.04858203072293	1.35899127631024	0.00742620195389444
STC1	0.096837195341802	1.10168100022131	0.981281348695747	1.23685325096803	0.101014379923527
MT2A	-0.0824804353428026	0.920829453393163	0.834120439835026	1.01655209696582	0.102129572069046
CST3	0.199474018574925	1.22076049192171	1.05553281189418	1.41185206356837	0.0071806675559779

### 3.7 Ubiquitination−related prognostic genes in GBM

**Figure 7:**
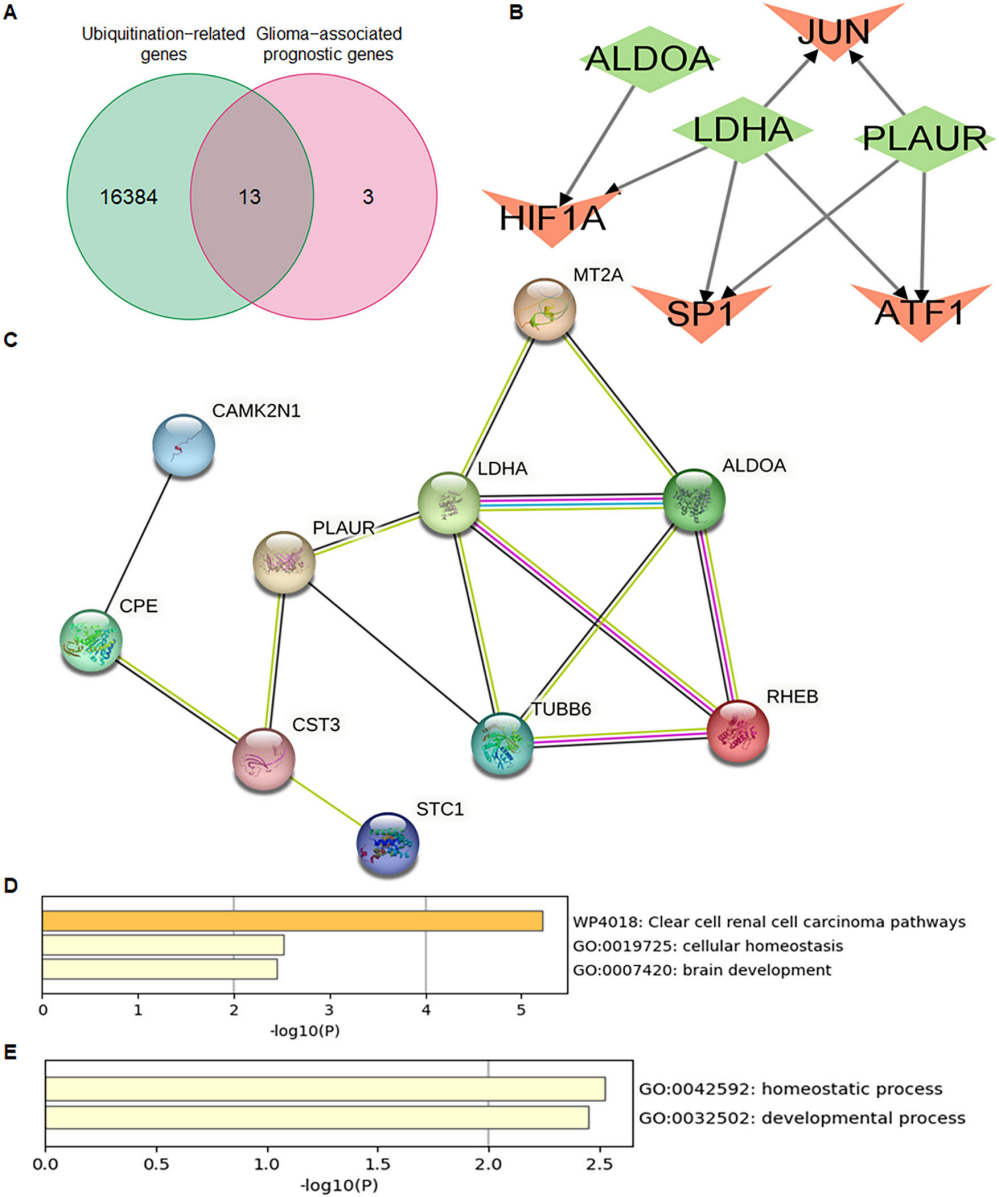
Identification and characterization of ubiquitination-related Prognostic genes in GBM. **(A)** Venn diagram showing the overlap between ubiquitination-related genes (URGs) and glioma-associated prognostic genes (GAPGs), yielding 13 intersecting UR-GAPGs. **(B)** Transcription factor (TF) analysis of the 13 UR-GAPGs, highlighting shared TFs such as JUN, HIF1A, SP1, and ATF1. **(C)** Protein–protein interaction (PPI) network of the 13 UR-GAPGs constructed using the STRING database (interaction score ≥ 0.15; disconnected nodes hidden). **(D)** Bar graph showing enriched biological terms across the input gene list, colored by p-value. **(E)** Gene Ontology (GO) analysis highlighting the top-level biological processes associated with the 13 UR-GAPGs.

A total of 16 glioma-associated prognostic genes and 16,384 ubiquitination-related genes (URGs) were obtained from the GeneCards database. The intersection of these two gene sets identified 13 ubiquitination-related glioma-associated prognostic genes (UR-GAPGs): LDHA, TUBB6, ALDOA, RHEB, MT2A, CST3, CDK5RAP2, VKORC1, NNAT, CAMK2N1, PLAUR, CPE, and STC1 (**Figure 7A**). Transcription factor analysis revealed that three of these genes—ALDOA, LDHA, and PLAUR—are co-regulated by transcription factors JUN, HIF1A, SP1, and ATF1 (**Figure 7B**). For the PPI network, a minimum interaction confidence score of 0.15 (low confidence) was set, and disconnected nodes were excluded, resulting in a network of 10 genes (**Figure 7C**).

### 3.8 Functional characteristics of ubiquitination-related prognostic genes

**Figure 8:**
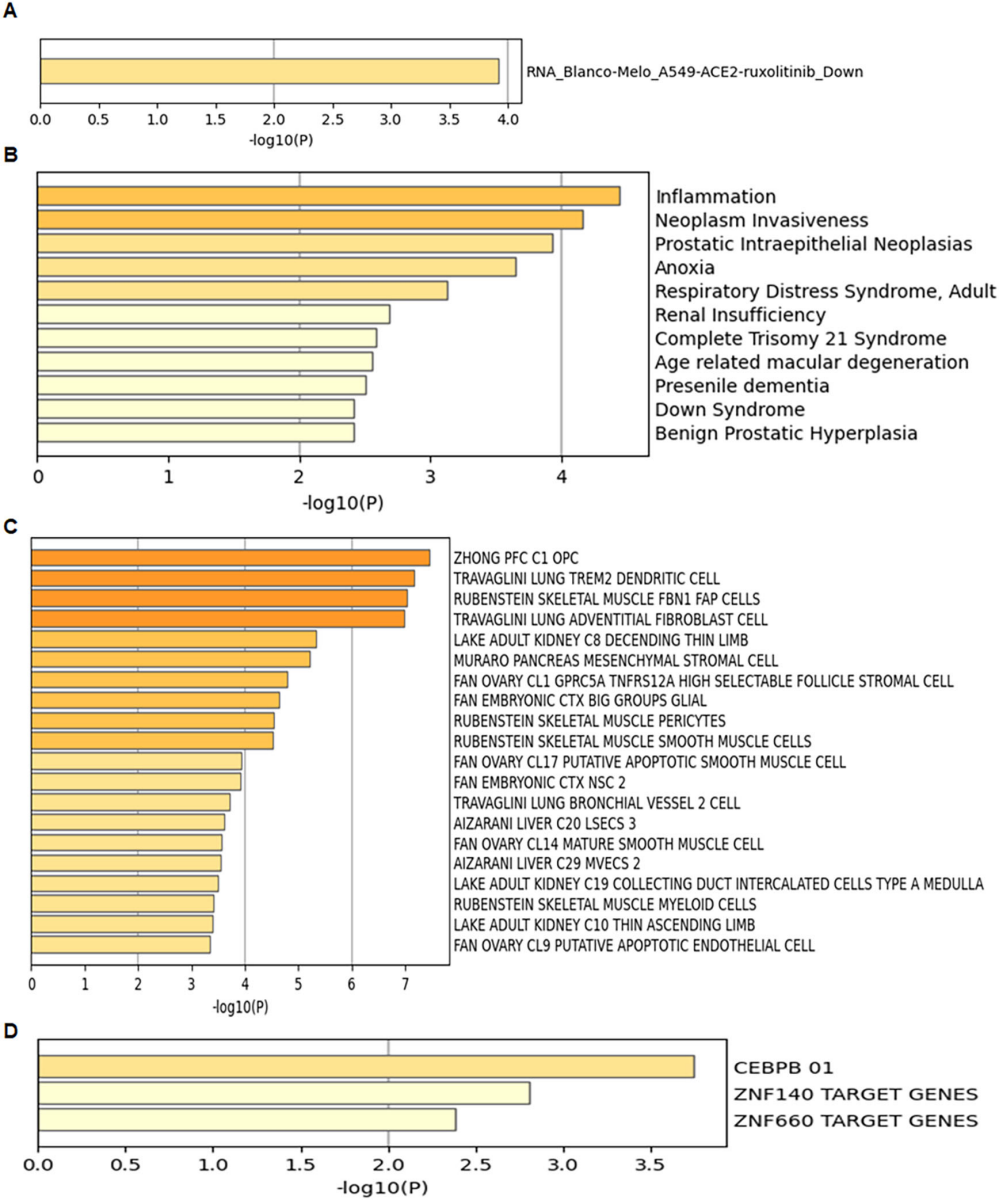
Enrichment analysis of the 13 UR-GAPGs across various databases. **(A)** COVID-19-related enrichment analysis. **(B)** Disease association analysis using the DisGeNET database. **(C)** Cell type-specific enrichment based on Cell Type Signatures. **(D)** Transcription factor target enrichment analysis identifying CEBPB_01 as the most significantly associated regulator.

Using the Metascape web portal, enrichment analysis of the 13 UR-GAPGs revealed that these genes are involved in pathways such as clear cell renal carcinoma, cellular homeostasis, and brain development (**Figure 7D**). Gene Ontology (GO) biological process analysis highlighted major roles in homeostatic and developmental processes (**Figure 7E**). Furthermore, a COVID-related enrichment analysis based on Blanco-Melo A549-ACE2-ruxolitinib RNA-seq data was conducted (**Figure 8A**). The DisGeNET database confirmed associations between these 13 genes and inflammatory processes, emphasizing the relevance of inflammation in their functional roles (**Figure 8B**). Analysis using the Cell Type Signatures database identified ZHONG PFC C1 OPC as the cell type most enriched for these genes (**Figure 8C**). Among transcription factor targets, CEBPB 01 emerged as the most significantly enriched (**Figure 8D**).

### 3.9 Differential expression and survival analysis of ubiquitination-related prognostic genes

**Figure 9:**
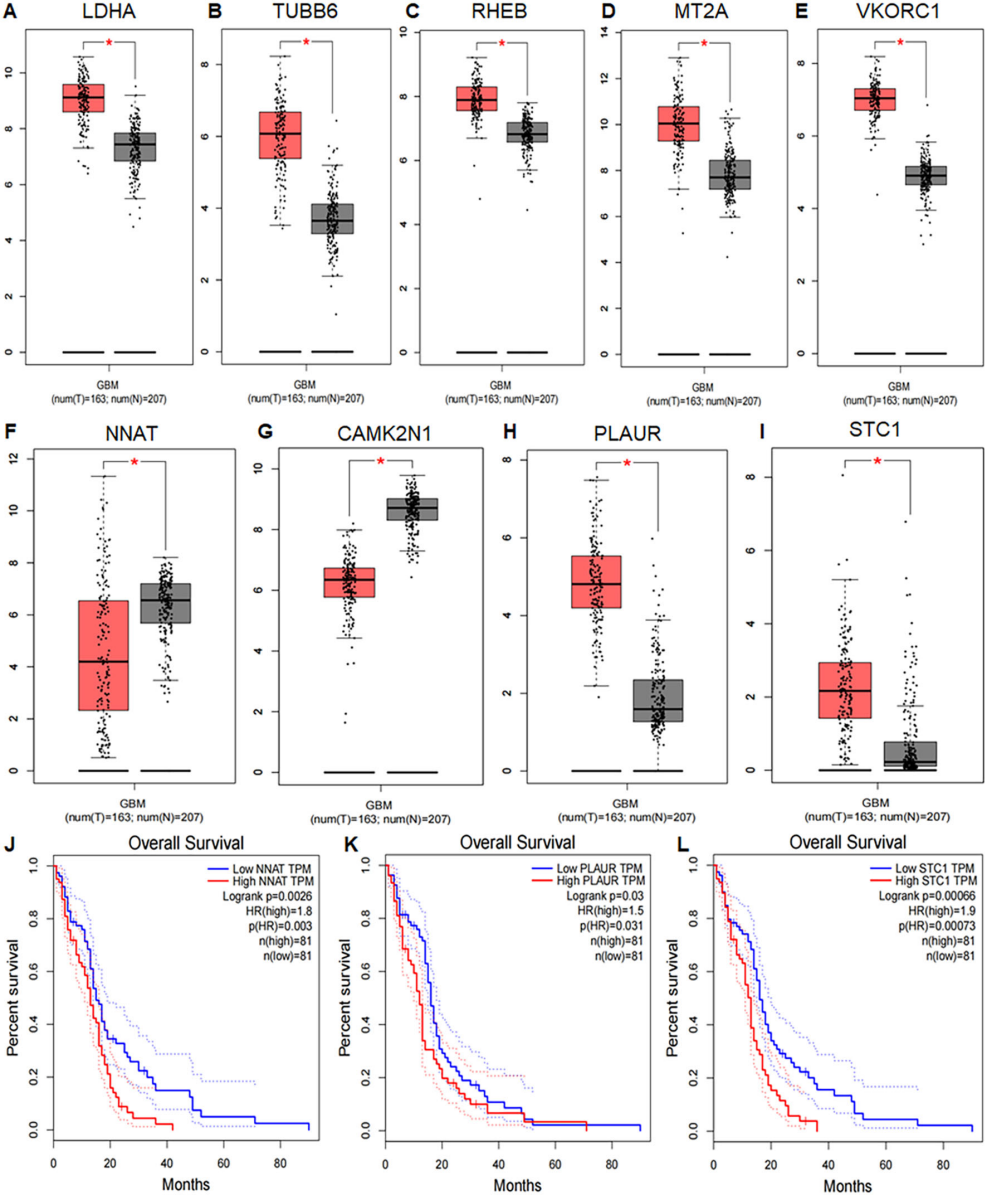
Differential expression and prognostic value of UR-GAPGs in GBM. **(A–I)** Box plots comparing gene expression between GBM tissues (TCGA) and normal tissues (GTEx). Nine UR-GAPGs showed significant differential expression (*P < 0.05): **(A)** LDHA, **(B)** TUBB6, **(C)** RHEB, **(D)** MT2A, **(E)** VKORC1, **(F)** NNAT, **(G)** CAMK2N1, **(H)** PLAUR, **(I)** STC1. **(J–L)** Kaplan–Meier survival curves for three UR-GAPGs significantly associated with overall survival in GBM patients (*P < 0.05): **(J)** NNAT, **(K)** PLAUR, **(L)** STC1.

Differential expression analysis using the GEPIA database, which integrates data from TCGA and GTEx, included 163 GBM tumor samples and 207 normal tissue samples. Box plots showed that LDHA, TUBB6, RHEB, MT2A, VKORC1, NNAT, CAMK2N1, PLAUR, and STC1 were significantly differentially expressed between tumor and normal tissues (P < 0.05) (**Figures 9A–I**). For survival analysis, patients were divided into high- and low-risk groups based on the average expression levels of the 13 UR-GAPGs. Kaplan–Meier analysis demonstrated that NNAT, PLAUR, and STC1 were significantly associated with overall survival (P < 0.05), with lower expression levels corresponding to improved survival outcomes (**Figures 9J–L**).

### 3.10 Machine learning

**Figure 10:**
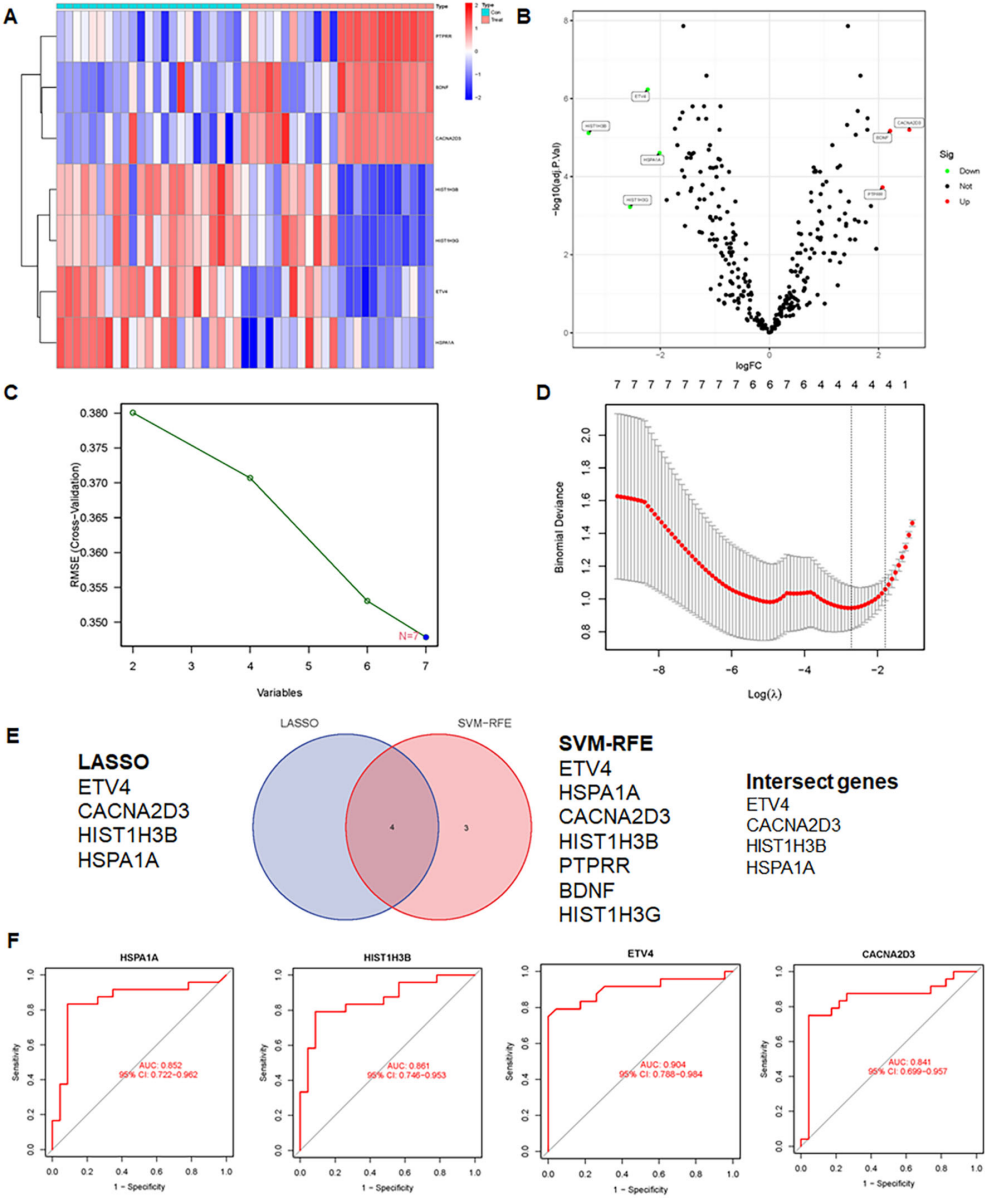
Integrative analysis and insights unraveling differential gene expression, feature selection, and intersection genes in GEO dataset. **(A)** Heatmap of differential expression genes in the GEO dataset. **(B)** Volcano plot of differential expression genes in the GEO dataset. **(C)** SVM-RFE results, presenting the important features or genes identified. **(D)** LASSO results, displaying selected features or genes with predictive power. **(E)** Venn diagram showing intersection genes identified by SVM-RFE and LASSO. **(F)** AUC of the training set.

**Figure 11:**
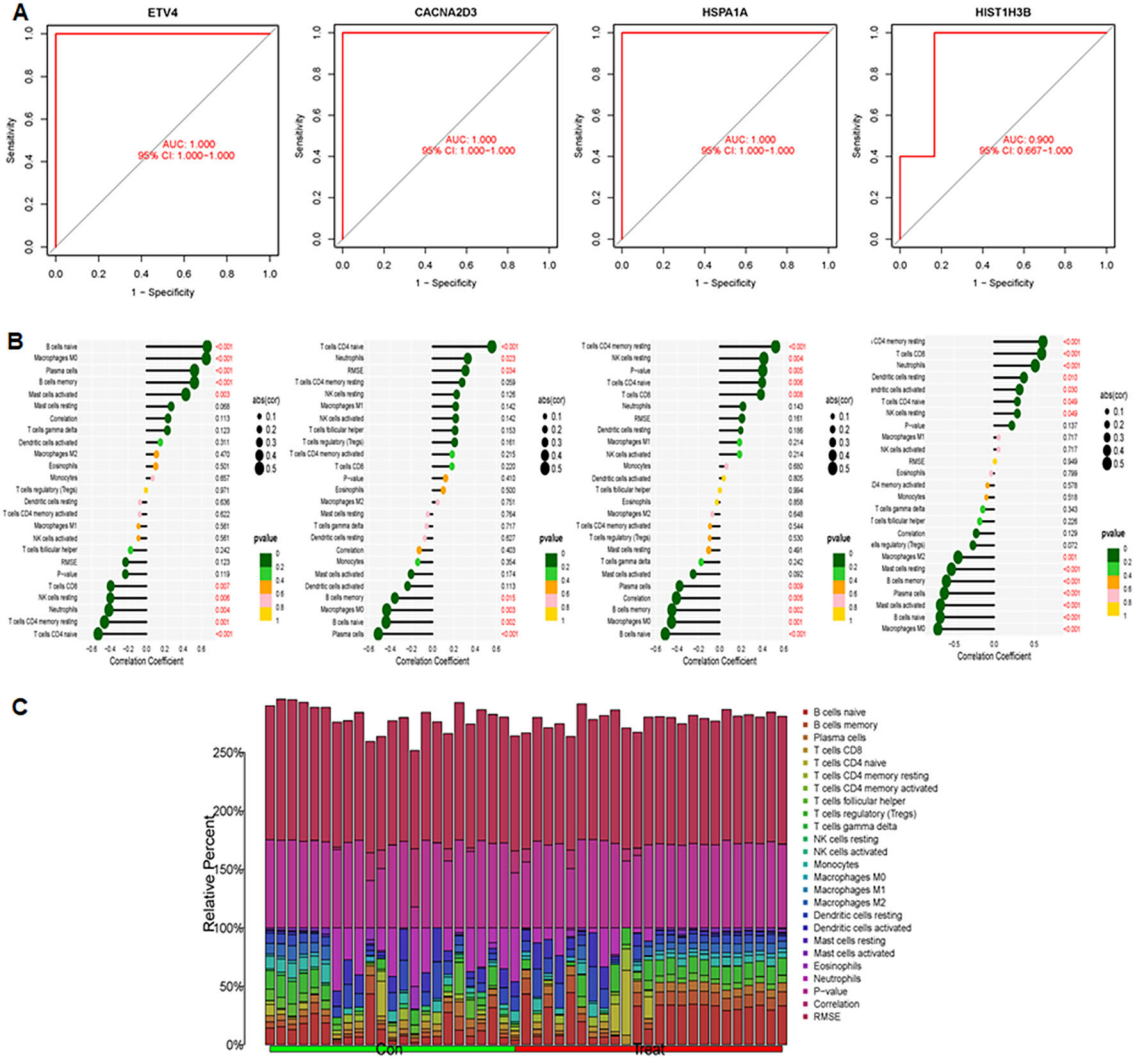
AUC performance and correlations between immune cells and four genes were explored. **(A)** Evaluation of AUC in the test set: examining AUC performance in the independent test set. **(B)** Correlation analysis between immune cells and four genes. **(C)** Infiltration patterns of 22 immune cell types, showing infiltration levels and patterns across immune cell subsets.

The study included two GEO datasets, GSE159605 and GSE186057, which were combined for differential analysis. The six most significantly different genes were visualized in a volcano plot and heatmap (**Figures 10A, B**). SVM-RFE, effective when there are more predictors than observations (33, 37), and LASSO were used to screen genes. The results of LASSO and SVM-RFE were displayed in **Figures 7C, D**. LASSO identified four key genes: ETV4, CACNA2D3, HIST1H3B, and HSPA1A (**Figure 10C**), while SVM-RFE identified seven genes: ETV4, HSPA1A, CACNA2D3, HIST1H3B, PTPRR, BDNF, and HIST1H3G (**Figure 10D**). The intersecting genes include ETV4, CACNA2D3, HIST1H3B and HSPA1A (**Figure 10E**).The combined data were divided into a training set and a test set to evaluate the sensitivity and specificity of selected biomarkers. In the training set, the AUCs of HSPA1A, HIST1H3B, ET4V and CACNA2D3 were 0.852, 0.861, 0.904 and 0.841, respectively (**Figure 10F**). Testing the biomarkers on the test set yielded AUCs of 1, 1, 1 and 0.900, indicating high accuracy (**Figure 11A**). The immune infiltration patterns for 22 cell types in the control group and treatment group are shown in **Figure 11B**.

### 3.11 Inhibition of ETV4 attenuates cell proliferation and invasion

**Figure 12:**
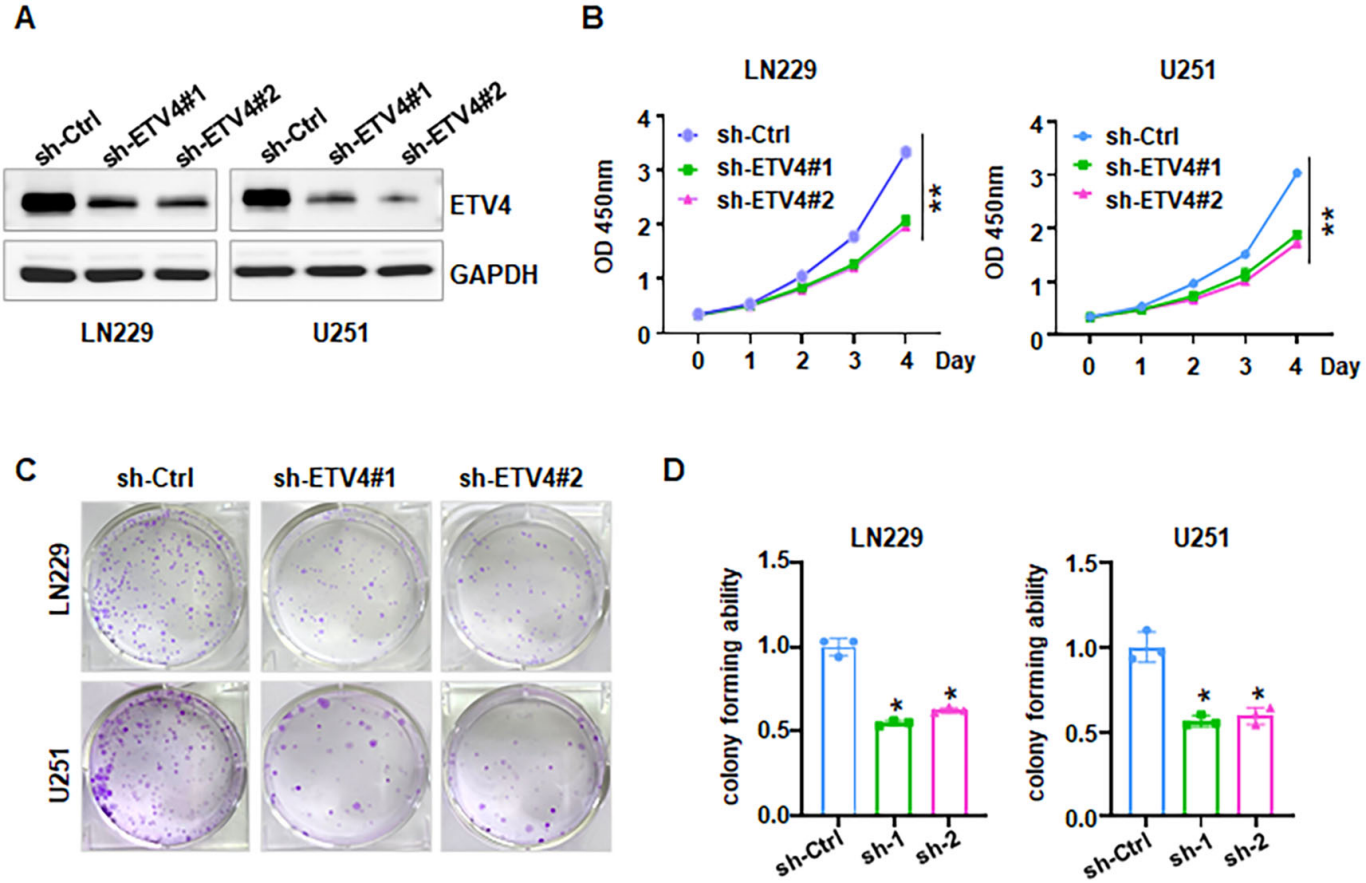
ETV4 knockdown inhibits cell proliferation in glioma cells. **(A)** Western blot analysis showed that shETV4 inhibited ETV4 expression. **(B)** CCK-8 assays showed shETV4 inhibited cell viability in glioma cells. **(C)** Colony formation showed that shETV4 inhibits cell colony formation. **(D)** Quantification of colony formation in glioma cells after shETV4 transfection. *P < 0.05; **P < 0.01.

**Figure 13:**
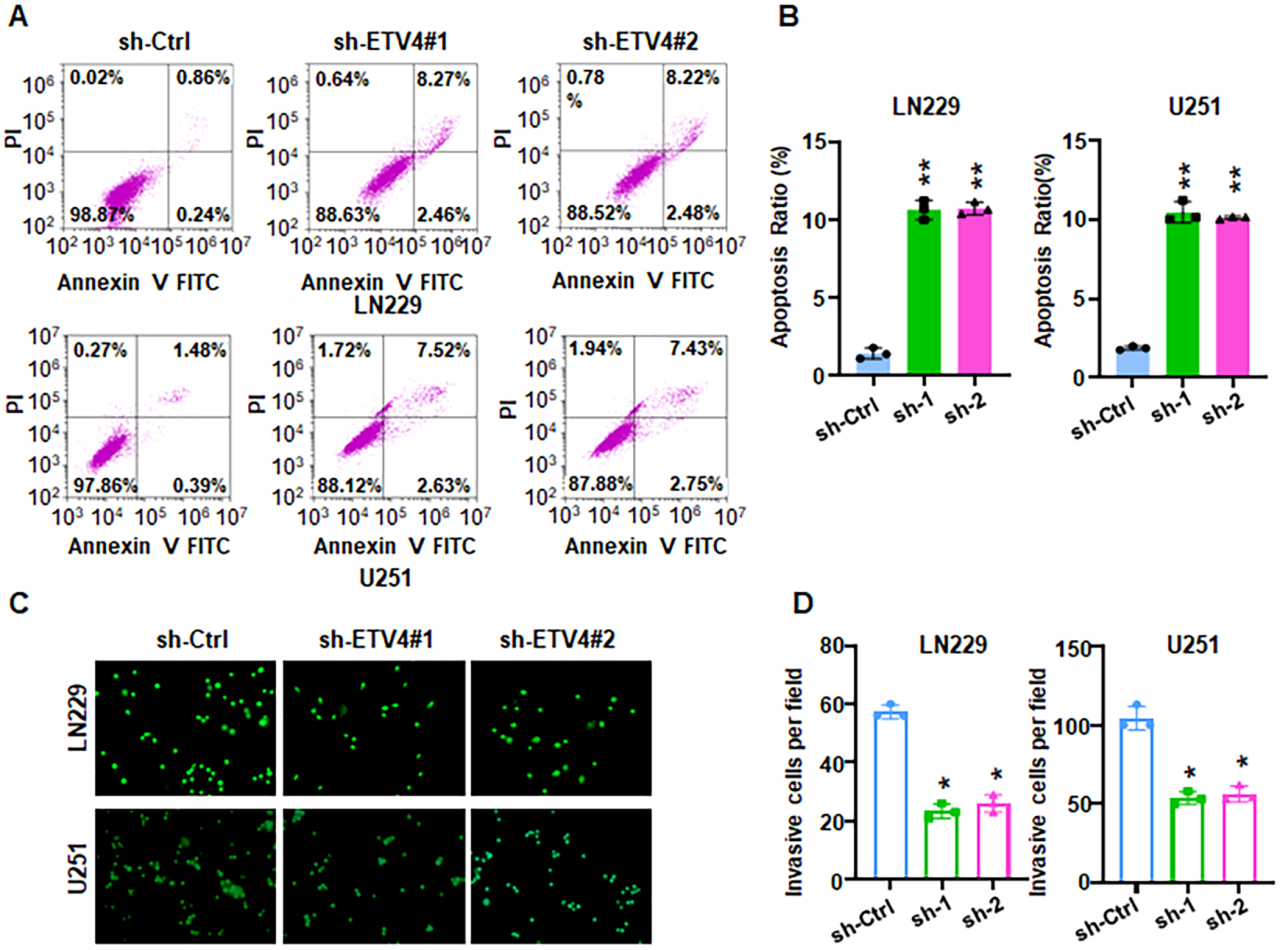
ETV4 knockdown increases apoptosis and reduces invasion. **(A)** Annexin V-FITC/PI staining showed that shETV4 increases cell apoptosis in glioma cells. **(B)** Quantification of apoptosis for panel **(A)**. **(C)** Transwell invasion assays showed that shETV4 reduced cell invasion in glioma cells. **(D)** Quantification of invasion for panel **(C)** *P < 0.05; **P < 0.01.

Evidence revealed that HSPA1A promotes tumor cell proliferation and invasion, contributing to the progression and recurrence of GBM (38). CACNA2D3 suppressed cell proliferation, migration and invasion in glioma (39). The prognostic impact of HIST1H3B/C mutations in diffuse midline gliomas varies depending on patient age (40). However, the role of ETV4 in glioma development and progression remains largely uncharacterized. To explore whether ETV4 regulates cell viability in glioma cells, shETV4 transfection was conducted in LN229 and U251 cells. Western blotting results demonstrated downregulation of ETV4 in shETV4-transfected cells (**Figure 12A**). CCK-8 assays were performed to evaluate cell viability in glioma cells after shETV4 transfection. Cell viability was decreased in shETV4-transfected cells compared with control group (**Figure 12B**). Moreover, colony formation results revealed that shETV4 transfection decreased colony formation ability in glioma cells (**Figures 12C, D**). Additionally, cell apoptosis was increased in shETV4-transfected glioma cells (**Figures 13A, B**). Strikingly, Transwell invasion assays showed that shETV4 transfection reduced cell invasive ability in glioma cells (**Figures 13C, D**). Hence, ETV4 downregulation inhibits cell viability, proliferation and invasion in glioma cells.

## 4 Discussion

The advent of single-cell sequencing has enabled high-throughput analyses of the genome, transcriptome, and epigenome at the single-cell level, exposing gene structure and expression profiles within individual cells and reflecting cellular heterogeneity (41–43). This approach has allowed researchers to explore putative monocyte oncogenes in gliomas, assess monocyte functional states across various phases, and examine intercellular communication channels using published single-cell sequencing data. Chen et al. employed a machine learning-based approach and identified UPP1 as a critical oncogene involved in tumorigenesis and immune evasion in gliomas (44). Similarly, Hu et al. integrated scRNA-seq with machine learning techniques to uncover the role of ALPK1 in shaping tumor immune heterogeneity and regulating the TGF-β signaling pathway in glioma (45). Integrating scRNA-seq with machine learning, Yang et al. revealed the heterogeneity of GBM-associated neutrophils and developed a prognostic model based on VEGFA-expressing neutrophils (46). In this study, single-cell sequencing data of GBM (GSE159416) was used to analyze three distinct cell differentiation states in glioma tissues, with most of the 14 enriched clusters being associated with fibroblasts. Marker genes for each of these cell states were combined to form DRGs. To predict the prognosis of glioma patients, a prognostic model based on 16 DRGs was developed.

The AUC values of our model were higher than those of other glioma prognostic models, suggesting greater accuracy in predicting the prognosis of glioma patients. By means of LASSO and SVM-RFE machine learning, four disease-associated genes, including ETV4, CACNA2D3, HSPA1A and HIST1H3B, were identified. These genes demonstrated high sensitivity and specificity in both training and test groups, with AUC values exceeding 0.9. ETV4 is a member of the polyoma enhancer activator 3 (PEA3) family, which plays an important role in cell growth, invasion and metastasis (47). During embryogenesis, ETV4 is widely expressed in various tissues, promoting the morphogenesis of epithelial-derived organs such as the kidneys (48), lungs (49), and breasts (50). However, in adults, ETV4 is rarely expressed in normal tissues, mainly appearing in tumor tissues such as those in breast, gastric, prostate, colon and ovarian cancers. Numerous studies have shown that high expression of ETV4 in prostate cancer (51), breast cancer (52), colorectal cancer (53), pancreatic cancer (54), and cholangiocarcinoma (55) often correlates with poor prognosis. Notably, one study showed that ETV4 expression increases with glioma grade progression (56). ERK kinase promoted phosphorylation of ETV4, leading to blockade of ETV4 ubiquitination and degradation in colorectal cancer (57). PTK6 induced phosphorylation of ETV4 and increased nuclear translocation of ETV4, leading to enhanced metastasis in bladder cancer (58). We found that inhibition of ETV4 attenuates cell proliferation and invasion in glioma cells.

CACNA2D3 is an auxiliary member of the α-2/δ subunit triple family with voltage-dependent calcium channel complexes and plays a key role in tumor suppression. CACNA2D3 is downregulated in gliomas and functions as a tumor suppressor (39). This downregulation in glioma cells and high-grade glioma tissues is associated with increased methylation (39). Additionally, a case report indicates that HIST1H3B K27 mutation is associated with glioma development of (59–61). Genomic analysis of HIST1H3B mutations may aid in timely glioma diagnosis, supporting surgical and clinical management of these patients (61). In gliomas, heat shock protein 70 (Hsp70, HSPA1A) is overexpressed in the cytoplasm (62). Overexpression of HSPA1A significantly enhances cell proliferation, with cellular immunofluorescence revealing its primary localization in the cytoplasm, where it promotes tumor cell proliferation (62).

For ubiquitin-related biomarkers, LDHA-mediated metabolic reprogramming has been shown to promote cardiomyocyte proliferation by reducing reactive oxygen species (ROS) and inducing M2 macrophage polarization (63). In clinical tissue samples, PTMs were quantitatively assessed, highlighting the pivotal role of ALDOA K330 ubiquitination/acetylation in tumor progression (64). RHEB ubiquitination plays a regulatory role in growth factor-induced activation of mTORC1 (65), and the Cullin3-Rbx1-KLHL9 E3 ubiquitin ligase complex has been shown to mediate RHEB ubiquitination, thereby facilitating amino acid-induced mTORC1 activation (66). Additionally, Malvidin has been found to reduce trauma-induced heterotopic ossification of tendons in rats by promoting RHEB degradation via the ubiquitin–proteasome pathway (67). In GSH-depleted RAW 264.7 cells, hydrogen peroxide triggers Beclin 1-independent autophagic cell death through suppression of the mTOR pathway via ubiquitination and degradation of RHEB (68). Moreover, STC1, a ubiquitin-related gene, is significantly upregulated in lung adenocarcinoma and is associated with poor prognosis, suggesting its potential as a biomarker for prognosis evaluation, tumor characterization, and therapeutic decision-making (69). However, despite these findings, it remains unclear whether these ubiquitin-related biomarkers play a role in glioblastoma (GBM). Their potential as novel therapeutic targets or biomarkers for precision medicine in GBM warrants further investigation.

## 5 Conclusions

In conclusion, our prognostic model based on differentiation-related gene signatures shows promise for predicting glioma prognosis and immunotherapy response. Furthermore, the characterization of ubiquitination-related features and machine learning–identified biomarkers provides novel insights into GBM progression, diagnosis, and treatment. Our study has several drawbacks. First, this research is based on bioinformatics analysis, and experimental validation is needed to further explore the underlying molecular mechanism. Our data is sourced from the TCGA and GEO databases. Therefore, validating the findings using independent clinical cohorts or third-party datasets is crucial to enhance the robustness and reliability of the results. There is a lack of experimental validation to support the transcriptomic predictions of immune infiltration, such as flow cytometry or immunohistochemical (IHC) staining of immune cell markers. All immune profiling in the study is based solely on transcriptomic data, with no spatial information to determine whether immune cells are excluded, infiltrating, or peripherally localized within the tumor microenvironment. Moreover, the clinical utility of immune signatures could be strengthened by correlating gene expression profiles with treatment responses in cohorts receiving immune checkpoint blockade therapy. In addition, the glioma cell lines LN229 and U251 both harbor p53 mutations, which may limit their ability to represent the broader biological heterogeneity of glioma. Orthotopic or subcutaneous GBM xenograft models should be employed to validate the *in vitro* findings and assess their relevance *in vivo*. Lastly, the functions of CACNA2D3, HIST1H3B, and HSPA1A in GBM should be validated through both *in vitro* and *in vivo* experiments. Collectively, we have constructed a novel GBM-related model to assess patient prognosis and identified four new signatures for diagnostic prediction, which may prove beneficial for future treatment strategies in GBM patients.

## Data Availability

The raw data supporting the conclusions of this article will be made available by the authors, without undue reservation.
